# Social studying and learning among medical students: a scoping review

**DOI:** 10.1007/s40037-017-0358-9

**Published:** 2017-05-17

**Authors:** Daniela Keren, Jocelyn Lockyer, Rachel H. Ellaway

**Affiliations:** Cumming School of Medicine, University of Calgary, Calgary, Alberta Canada

**Keywords:** Social studying, Undergraduate medical education, Study groups, Small group learning, Scoping review

## Abstract

**Introduction:**

Medical students study in social groups, which influence their learning, but few studies have investigated the characteristics of study groups and the impacts they have on students’ learning. A scoping review was conducted on the topic of informal social studying and learning within medical education with the aim of appraising what is known regarding medical student attitudes to group study, the impact of group study on participants, and the methods that have been employed to study this.

**Methods:**

Using Arksey and O’Malley’s scoping review principles, MEDLINE, EMBASE and CINAHL were searched, along with hand-searching and a targeted search of the grey literature; 18 peer reviewed and 17 grey literature records were included.

**Results:**

Thematic conceptual analysis identified a number of themes, including: the nature of group study; the utility and value of group studying including social learning facilitating student engagement, social learning as a source of motivation and accountability, and social learning as a source of wellbeing; and student preferences related to group studying, including its homophilic nature, transgressiveness, and effectiveness. Despite these emerging factors, the evidence base for this phenomenon is small.

**Discussion:**

The findings in this scoping review demonstrate a clear role for social interaction outside of the classroom, and encourage us to consider the factors in student networking, and the implications of this on medical students’ academics. We also highlight areas in need of future research to allow us to better situate informal social learning within medical education and to enable educators to support this phenomenon.

**Electronic supplementary material:**

The online version of this article (doi: 10.1007/s40037-017-0358-9) contains supplementary material, which is available to authorized users.

## What this paper adds

Some medical students form informal social study groups, which may influence academic outcomes and facilitate learning. Important themes identified with regards to social study include the nature of social study, the utility and value of social study, and student preferences with regards to social studying. Social learning can be a source of motivation, accountability, support and well-being. It may be homophilic and transgressive in nature. Students have varied perceptions regarding its effectiveness. However, the evidence for this phenomenon is small. An understanding of social studying and its effects on students’ academics may allow medical educators to better support this phenomenon.

## Introduction

Some medical students form informal social study groups while others do not [1]. There is evidence that indicates that these groups can influence exam results [2] and facilitate learning and sharing of resources [1]. The formation of these social groupings has been shown to be influenced by a number of factors including gender and ethnicity [2], and these groups are more likely to be a source of information gathering and exchange than formally assigned learning groups [3–5]. Student learning communities are thought to positively impact medical students’ social and educational relationships [6]. Although social studying would seem to play an important role within medical education, it seems to have received relatively little attention. Our starting point for this scoping review was to appraise what was known on this topic as a precursor to conducting our own research. The review objectives were to: identify what has been published regarding medical student attitudes to group study, the impact of group study on participants, and the methods that have been employed to investigate informal social studying and learning among medical students. We were also interested in identifying gaps in the literature to inform our research strategies. That social studying and learning is an area that is relatively under-researched in medical education is reflected in the absence of any common conceptual framework or terminology for this phenomenon. Therefore, for the purposes of this review, we used the following provisional definition of social studying and learning: any independent, self-directed and self-organized approach to learning or studying that involves students working with their peers for the purposes of study, learning, or revision. It is important to note that this is distinct from group activities that are organized for students as part of their coursework. We report on the review here using the PRISMA framework [7].

## Methods

We selected Arksey and O’Malley’s scoping review methodology [8] to capture the breadth of existent knowledge on the subject of medical student informal social study while being relatively inclusive regarding study methodologies and sources. This methodology is particularly useful for topics that have not been extensively explored, or for which many different study designs and contextual approaches have been used [8]. This review presents a synthesis of results from two separate searches:

1. a search of the peer-reviewed and published research to date and
2. a search of online ‘grey literature’ sources.

### Eligibility

#### Peer-reviewed published records.

Studies were eligible for review if they considered one or more aspects of social studying and learning as per our provisional definition. As the purpose of the scoping review was to learn about the nature of student-driven group learning, group learning that was a mandatory part of a program or course and was facilitated by a teacher or instructor was not considered. Studies could have employed any methodological approach but they had to have been published in English after 1995 to be eligible.

#### Grey literature.

Anticipating that students may describe or discuss their own experiences and that medical schools may provide guidance to students online, we also considered grey literature sources as long as they reflected medical student thinking or advice to medical students regarding informal group study. For the purposes of this review, grey literature was defined as any online sources that had not been peer-reviewed. Sources included student blogs, medical school websites, and online discussion forums. Grey literature sources also needed to be publicly accessible web pages or sites published in English. No date range was specified for these sources.

### Information sources

#### Peer-reviewed published records.

We targeted MEDLINE, EMBASE, and CINAHL databases for this review. We piloted various search options and finally settled on a search by title and abstract using the search terms: [[“medical student” OR “medicine” OR “medical education”] AND [“collaborative learning” OR “informal collaborative learning” OR “cooperative learning” OR “informal cooperative learning” OR “social study(ing)” OR “informal social study(ing)”, OR “informal group study(ing)” OR “informal group learning” OR “social learning”]]. The 60 most recent studies that were retrieved using this search were screened by title and abstract to evaluate the efficacy of the search strategy used. As less than ten of the studies were applicable, we expanded our trawl by conducting three further searches using:

- [“education/medical/undergraduate”] AND [“social learning” OR “peer group” OR “cooperative learning” OR “collaborative learning” OR “peer learning”] AND [“qualitative research”],
- [“education/medical/undergraduate”] AND [“social support”], and
- [“medical students”] AND [“social networks”] AND [“UK”].

Once we had screened for eligibility we then scanned the reference lists of those papers that had met the inclusion criteria for additional sources not identified by the search. All findings were exported to EndNote, where duplicates were deleted. We also conducted manual searches of five contemporary medical education textbooks [9–12] but found no chapters or sections that met our inclusion criteria.

#### Grey literature.

Because we were interested in students’ perspectives and informal discussion on the topic, we performed a targeted search of the grey literature by searching Google using the terms: “medical students study groups”, “medical student study strategies”, and “how to study in medical school.” All sources from the first 10 Google pages were screened for eligibility based on their discussion of social studying and learning. This screening approach was taken because links appeared to no longer be relevant after 10 pages of Google results.

### Study selection

#### Peer-reviewed published records.

We first reviewed the title and abstract of studies from the database searches. We then undertook a full-text review of the items retained from the first stage. Items were retained only if they considered social studying and learning, as per our stated definition, among undergraduate medical students.

#### Grey literature.

We first screened the link titles for each Google search and then undertook a full-text review of all items retained from the title screen. As with the peer-reviewed literature, items were retained if they considered social studying and learning in undergraduate medical education.

### Data extraction

We developed a data abstraction instrument and iteratively revised it based on its use on a random sample of ten items from those selected for review. The final instrument included items for study characteristics (e. g. year of publication, country where research was conducted), characteristics related to the methods, study findings, and comments regarding positioning of the study relative to the research questions for this review. This instrument was applied to both the peer-reviewed and grey literature.

### Synthesis of results

Extracted data were qualitatively synthesized [13] using thematic coding techniques, with reviewers working first individually and then collaboratively to identify recurring themes and to suggest middle-range theories [14]. Provisional findings were discussed and iteratively developed among the study team. The lead reviewer [DK] was a medical student at the time of the study and was supported by two experienced qualitative researchers [RE and JL] in undertaking this synthesis.

## Results

### Study selection

**Fig. 1:**
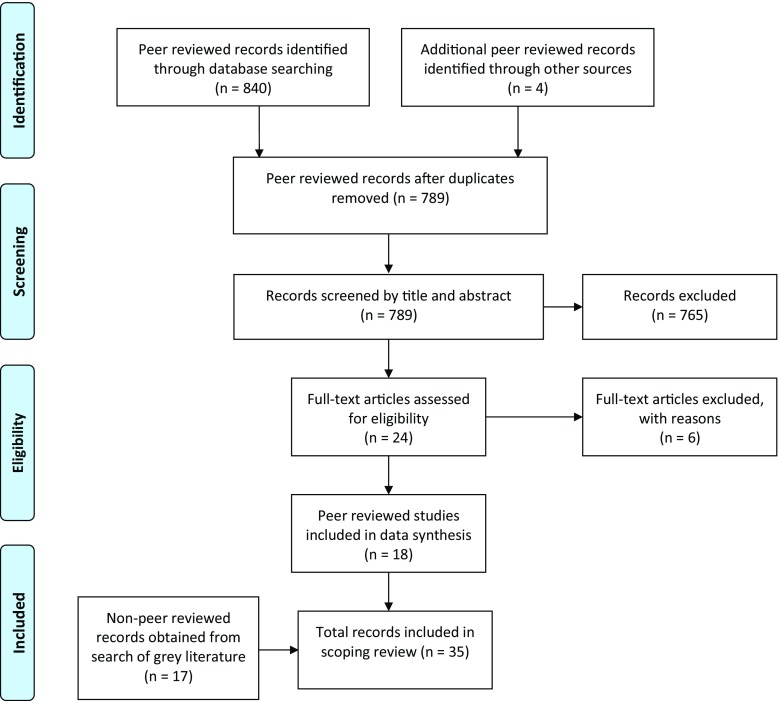
Flowchart of the data collection and study selection process in a scoping review of the literature on informal group studying and learning in medical education. (Created in accordance with PRISMA guidelines for transparent reporting of systematic reviews [7])

The initial database search identified 840 items, from which 55 duplicates were removed. Three additional records were identified from scanning the reference lists of relevant papers. The search of the grey literature also identified an additional peer-reviewed published article. Screening titles and abstracts for eligibility within the parameters for the review left 24 eligible items. Screening full-text for eligibility left 18 items. The search of the grey literature identified 17 non-peer reviewed sources for review. The PRISMA [8] diagram for the review is outlined in Fig. 1.

### Study characteristics

An overview of the characteristics of the sources reviewed is presented in Table 1 and details of study methods and inclusion of student voices are presented in Table 2, which are presented as Online Supplementary Material. We identified seven core topics in the 18 peer-reviewed studies included in this review: the influence of social learning on academic outcomes, the role of group studying and learning in medical education, understanding social networks, student wellbeing, ethnicity achievement gaps, academic use of social media, and the nature of group learning in medical education. The peer-reviewed literature tended to consider elective collaborative learning alongside investigation of social networking and ethnicity gaps in medical education, academic outcomes, student well-being and social media use. There were six studies that explicitly investigated the nature and roles of informal group learning in medical school.

Of the 17 grey literature sources, 14 took the form of opinion articles, and three were social media websites or forums. Interestingly, just over half of these sources (9) did not contain any student voices. Three of the sources were exclusively composed of student voices, and another three were extensively composed of student voices. One source included minimal student voices and another did not specify whether or not it included any student voices. Some grey literature sources aimed to advise medical students on how to study most effectively and others were online discussions of learning strategies and what works for students. Two sources focused on advising students on how to effectively use study groups [15, 16] and two described the relative advantages and disadvantages of study groups [17, 18]. Grey literature sources were inclusive of many different kinds of informal learning, and they solely reflected the voices of those who had participated in the conversations. Forums and online discussions demonstrated a variety of opinions regarding group learning, with some students finding it very valuable while other students preferred and/or recommended independent learning as a superior approach. Grey literature sources that were part of medical school websites tended to advise students how to make use of group learning and studying.

#### Synthesis of results.

The thematic conceptual analysis identified three overarching themes related to research in informal group studying in medical education.

### The nature of group study

In the peer-reviewed literature, some sources described the characteristics of the students who engage in and benefit from group study. For example, one study suggested that students tended to form groups with individuals with similar personalities to their own [19]. This study also found informal study groups to be effective when they were supportive, socially cohesive, trusting and loyal [19].

The general characteristics of effective social studying and learning were more commonly considered in the grey literature sources. These suggested that group study was best suited for revising previously learned material through students testing each other, reviewing notes together, and clarifying points of uncertainty [1, 20–24]. Grey literature sources also supported the idea that like-minded individuals work well together [23, 25]. Sources also described group study as only effective when members are organized and maintain group structure and focus [15, 26].

### The utility and value of group study

This was reflected in the following three subthemes:

#### Social learning facilitating student engagement.

Boysen et al. [27] described a situation where, although fewer than one-third of the class was present for any given lecture, the class averaged in the 90^th^ percentile on their national exams because of their use of informally organized peer-to-peer learning. Network analysis of formal and informal group studying identified students’ self-selected personal academic support networks as often being of more value in ‘activities important to their academic success’ [5]. Social studying has been shown in the published literature to provide an opportunity for students to draw on their peers to help to fill gaps in their own knowledge [19, 28].

Grey literature sources suggested that informal group study can make learning more active and engaging [17, 21, 23, 26, 29, 30], both through checking one’s understanding of concepts, and by teaching peers [20, 21, 30, 31]. This suggests that informal group learning may help students to become more active and engaged in their learning during their independent study time.

#### Social learning as a source of motivation and accountability.

The peer-reviewed literature showed that students tended to influence each other’s study habits when they compared and critiqued their own motivation and dedication with that of their peers [1], which in turn increased student motivation [1]. Studies also demonstrated that discussions with peers can help students to gauge the strengths of and identify deficits in their skills and knowledge [19, 32]. This observation was further supported by the grey literature, which suggested that students studying on their own, but co-present with their peers can also be motivating [20–23].

#### Social learning as a source of support and wellbeing.

The peer-reviewed literature described social nature of group studying as providing students with emotional support [1, 33–35]. For instance, a study exploring the relationship between medical student group membership and well-being found that group membership creates a platform for individuals to give and receive social support [35]. Mechanisms for this included a sense of mutual trust among group members [35]. The role of group study in helping medical students cope with the academic demands of their programs was another recurring theme. For instance, students perceived to be ‘at risk’ of failing their summative exams participated in study groups led by more senior students, which helped them adjust to the demands of medical school as well as addressing their more immediate academic challenges [34]. Groups may also study collaboratively using an online platform such as Facebook to share their experiences and to provide support to each other [33]. A study of peer groups in clerkship suggested that these groups can be a source of social support for learners transitioning into new clinical contexts [32].

### Students’ preferences with regards to group studying

We encountered significant variability in student preferences with regards to social learning and studying (see Table 1 of the Online Supplementary Material). Both the published literature and the grey literature contained a diversity of opinions with regards to how student groups should be organized, how they should be conducted, and even whether they should be used at all. This is reflected in the following three themes:

#### Homophilic social learning.

In the peer-reviewed published literature, several studies noted that students tend to study and socialize with students who are similar to them [2, 3]. In one study, although ethnic minority exclusion from groups was not seen, there was an underrepresentation of ethnic minorities in the largest network component [3]. Moreover, Woolf et al. showed that students who did well academically were more closely linked in the social network to each other, with students who performed poorly being more closely linked to each other [2]. These homophilic tendencies may create asymmetrical opportunities for medical students to succeed academically. Two studies included in this review assessed social learning with respect to ethnicity-dependent attainment gaps in medical education [5, 36]. Vaughan et al. [5] found that students’ self-selected personal academic support networks were homophilous by ethnicity, particularly among the dominant ethnic group. Although students with non-dominant ethnic backgrounds more often achieved lower grades, network factors had a greater impact on achievement than ethnicity or religion alone [5]. Woolf et al. [36] showed that univariate ethnic differences were found based on learning styles, living at home, and first language.

#### Transgressive social learning.

The peer-reviewed literature suggested a recurrent theme that group study was favoured by students when it did not involve faculty and when it was not mandatory or imposed on them [32–34, 37]. It was notable that student exam scores did not differ between those who did participate in mandatory sessions and those who did not [30]. Autonomy and self-regulation of peer learning may be valued because it creates a space for students to discuss topics of their choosing without fear or concern regarding faculty impressions or opinions [32, 33]. For instance, peer groups can be a way of learning the implicit rules of clerkship, or sharing expectations that may not be made explicit by faculty [32]. Learning the hidden curriculum illustrated the kinds of transgressive behaviours that students may engage in [33].

#### Limits to the effectiveness of social learning.

Although our review identified many potential benefits of informal social learning, there were also countervailing voices in the peer-reviewed literature that argued that group studying was of little or no value to medical students and that solitary study is more efficient than group study [28]. As a participant in the study by Dick [28] noted: *‘… we just got so confused and we were just going in tangents, it’s literally like the blind leading the blind’*. Hendry et al. demonstrated that group study may distract students from their learning [19].

This was supported by the grey literature, in which some students expressed the opinion that group studying is not an effective learning strategy [23, 38, 39], group study is described by some students as having the potential for students to distract each other from their learning [17, 40] and, by comparing themselves to each other, it can contribute to additional stress [23]. Some students may try to dominate the group or to show off, which may also negatively impact learning [15, 17, 40].

It was notable that group study was not ubiquitously valued by medical students or medical educators. This may be attributed to differences in learning styles, or socialization, and should be considered when examining the phenomenon of independent collaborative learning in medical education.

## Discussion

Our review identified 18 peer-reviewed sources published since 1995 and 17 grey literature records on social studying and learning in medical education, and is the first review (as far as we can tell) to investigate social studying and learning among medical students. In the modest body of literature that we identified, it was clear that self-directed informal group learning is quite common (although not ubiquitous) within medical education, and that it can serve different purposes. Although it is undertaken outside of official program precincts, a better understanding of medical students’ approach to social study would seem to be an important issue. For instance, better understanding of informal social study may enable medical educators to support or guide their learners in this regard. Indeed, it was clear, given the number of articles from medical school websites that we found [20, 21, 26, 29, 31], that many medical educators are already providing guidance to students regarding whether or not to, and how to, study socially.

We identified a variety of primary outcomes of interest including several that directly focused on the role of group studying and learning in medical education [1, 19, 27, 28, 32, 37, 41]. Other studies investigated group learning as it existed within the context of social network formation [2, 3, 5] or in terms of its relationship with differences based on ethnicity and ethnic achievement gaps in medical schools [5, 36]. Some studies considered the effect of social studying and informal collaborative learning on academic outcomes [34, 42, 43] or on student wellbeing [1, 35]. Grey literature sources tended to focus on advising medical students regarding study strategies in general [20–26, 29–31, 38–40], within which a smaller number of sources focused on the advantages and disadvantages of group studying [16–18, 21].

We also found that a variety of methods were used to investigate informal social learning including: survey distribution, semi-structured interviews and focus groups (see Table 1 of the Online Supplementary Material). Only one of the studies included was experimental, in which mandatory informal peer-to-peer study groups were implemented in a medical school for students deemed ‘at risk’ of academic failure [34]. No randomized control trials have been completed on this topic, and there was limited use of intervention measures. The grey literature mostly consisted of opinion pieces on websites, blogs, and social media discussion forums [23, 25, 40]. Although we found a body of informally published and non-peer-reviewed literature on the topic, there are very few high quality studies investigating informal group studying in medical education.

The review identified several gaps in the current literature regarding informal collaborative learning and group studying within medical education. The most apparent gap was the disconnect between the grey literature and the published literature. All the sources included from the grey literature characterized group studying and learning, and many did so from the direct voices of learners themselves. This was rarely the case in the published literature. Few published studies directly sought to identify students’ perspectives on group studying and students’ self-expressed reasoning for engaging in this form of learning [1, 19, 28, 33]. Also, given the informal nature of group study, there was an apparent absence of student voices from the peer-reviewed literature on this topic. In addition, although many grey literature sources provided advice to students on the potential benefits and pitfalls of studying in groups, the published literature did not reflect a substantive evidence base to back this up, particularly with respect to academic outcomes. This suggests that we need more robust evidence regarding what constitutes good practice in group study, in particular regarding what kinds of group learning work better for what kind of learners in what circumstances. For example, although it was suggested, it was unclear whether the described benefits and value of social studying are different from the benefits of formal teaching (such as through lectures and small group learning). If this is the case, then it may well be that group study can fill gaps in students’ learning that cannot be filled using other approaches. If group study can provide something that formal programs do not, then we must ask whether it is the role of medical educators and medical schools to address this formally.

Our findings also suggest a degree of reciprocity among students when they study in groups. However, it may be that, when studying socially, some students give more of their knowledge and understanding to students who need it more. If this is the case, it poses the question of whether teachers in the formal curriculum are teaching to an entire classroom, or whether they are more likely teaching to a subset of the class who then go on to teach others in the informal group context. A better understanding of these asymmetries afforded by informal group learning may help educators better address this and support it in their programs. It was also clear that whether or not students engage in social learning, and how they do so, is highly variable. This suggests that a better understanding of the role of social learning on team dynamics and students’ growth as team players would be very valuable, as teamwork is such a critical component of a career as a physician. Finally, both the literature [2, 5] and DK’s personal experiences as a medical student suggested that informal study group formation may be influenced by homophilic bias, specifically with respect to gender and ethnicity, and that there are potential inequities of opportunity that should also be further explored.

We should note some key limitations to this review. Firstly, we identified a relatively small number of sources (*n* = 35) of which half were from the peer-reviewed literature. Although this highlights opportunities for future research in this area, it also limits the generalizability and reliability of our findings. We also noted that the studies conducted in particular medical schools may not generalize well as they involved small sample sizes, and idiosyncratic local circumstances. Within the grey literature, although it was clear that this topic is relevant to students and is well-discussed, our review could only include the material that has been discussed online and is publically accessible. We therefore lack the perspectives of those students who do not discuss their study and learning habits online, and the perspectives of those students who do engage in online forums may not reflect those of students in general. Finally, the grey literature described what students and educators believed to be effective with respect to social studying and learning, but we are unable to determine with certainty whether this was actually the case.

## Conclusion

This scoping review provides an important synthesis of the topic of informal social learning and studying within medical education. Our findings demonstrate a clear role for social interaction outside of the classroom, which obliges us to consider what factors are involved in the networking of students, and the implications that this may have on academic success in medical school. The results of the review allow us to better situate informal social learning within the broader context of medical education. The review also highlights the need for future research with the explicit purpose of improving our understanding of students’ motivations for studying socially, and their perceptions of the role of this learning tool in their education. For instance, if, as it seems, self-selected social grouping plays an integral role in the academic achievement of students who are engaging in it, then students who experience barriers in their ability to form these networks may be at a disadvantage. Our findings also indicate that a better understanding of how informal social learning impacts academic outcomes is needed. Ultimately, further research on this topic will better enable medical educators to support their students in their use of social learning as a learning tool.

## Caption Electronic Supplementary Material

Overview of the included studies

Characteristics of the reviewed sources
