# Supplementary material for: Social studying and learning among medical students: a scoping review
Source: Perspect Med Educ. 2017 May 17;6(5):311–8. doi: 10.1007/s40037-017-0358-9 (PMC5630528; doi:10.1007/s40037-017-0358-9)
Supplement: Supplementary file 1 — Overview of the included studies [file 40037_2017_358_MOESM1_ESM.docx]

*Table 1: Overview of the included studies*

| Author, Location and Reference Number | Aim | Methods and Medium | Participants | Main Findings |
| --- | --- | --- | --- | --- |
| Ali, 2016  England [33] | To identify and detail the specific kinds of educational activities that medical students partake in through Facebook, and to explore the use of student-created Facebook groups as a vehicle for enhancing learning | Focus groups  Semi-structured interviews  Thematic analysis | 24 medical students who self-identified as being Facebook users | Six common themes emerged: (1) collaborative learning; (2) strategic learning for preparation for assessment; (3) sharing experiences and providing support; (4) creating and maintaining connections; (5) personal planning and organization; (6) sharing and evaluating educational resources |
| Amgdad and Alfaar, 2014  Egypt [44] | To investigate the usefulness of integrating web 2.0 technologies into formal educational courses and modules | Survey | 176 undergraduate medical students | Most students agreed or strongly agreed that the following tools were useful: Dropbox for sharing papers, readings, and presentations; Google documents for sharing assignments and continuous evaluation; Facebook for communicating/discussion among course participants; email use for communication; Facebook events; Mandeley for group research projects; Youtube for sharing recorded lectures; live streaming of lecture; online registration application; online daily feedback system; google hangout for online meetings. |
| Anonymous, n.d.  USA [15] | To assist medical students in effectively using study groups as a study strategy | Opinion:  Article on medical school website | Not applicable – personal commentary | Study groups are described as effective only when organized, structured and focused. They should be made up of 2-6 individuals, and should ideally meet twice a week for 2 hours at a time. Agenda setting, rotating leadership and a formal note-taker were recommended. The emotional climate of study groups is addressed: avoiding anxiety is recommended, and the need to demonstrate intellectual superiority is discouraged in the context of group study. |
| Anonymous, n.d.  USA [21] | To provide medical students with strategies to improve their studying | Opinion:  Article on medical school website | Not applicable – personal commentary | Recommendations: include various different study/learning modes, study actively, repetition, use mnemonics and study aids (cards, note sheets), complement solitary studying with group studying (more expressive learning, assesses breadth of your knowledge, checks your understanding), don't base your willingness to engage on how much you like the course, use the faculty for help. |
| Anonymous, 2006.  USA [22] | To discuss efficient study strategies and what works for students | Discussion:  Online discussion forum | 29 unique participants (medical students, resident trainees, physicians) | Students recognize that study strategies are individual and the same strategies do not work for all students. Some suggestions include rewriting notes and repetition, making up your own study questions as you study, putting information into tables, getting together with others to talk about the material, studying with friends with compatible study habits, looking at the same information from a number of different sources, using old exams, using flashcards. Some have found study groups ineffective. |
| Anonymous, n.d.  USA [29] | To advise medical students regarding how to keep their studying more active | Opinion:  Article on school website | Not applicable – personal commentary | Look over practice questions before studying to know what information is relevant later. Make key aspects and relationships stand out in study notes. Switch activities to stay alert (ex switching material studied, or resource used). Finally, suggests using a study partner to take turns asking each other questions and talking through material - way to practice applying what you know. |
| Anonymous, n.d  Unspecified region [40] | To provide avenue for students/trainees/MDs to share how to successfully study | Opinion:  Online discussion forum | 13 unique participants (medical students, resident trainees, physicians and unspecified) | Many different opinions on how to study (in isolation, in groups, with self tests, etc.). Some key suggestions: organization, priorities, minimizing distraction, good health/hygiene, 'read, collate, create, teach,' learning to understand and not to memorize. Pros regarding group studying: ability to teach others and thus reinforce knowledge, efficiency of learning. Cons regarding group studying: members who are unprepared, show off, or become easily distracted. |
| Anonymous, 2012  USA [38] | To provide advice to medical students to help them in their studies. | Opinion:  Blog article | Not applicable – personal commentary | Recommendations: don't study last minute, avoid study groups because is not effective use of time, minimize distractions, do practice tests. Don’t be hard on yourself, learn about medicine outside of schoolwork, recognize that you are still a student and not yet a physician, set aside time to relax and be in touch with your family. |
| Anonymous, n.d.  USA [31] | To provide medical students with techniques for learning/studying in medicine | Opinion:  Article on medical school website | Not applicable – personal commentary | Recommendations: 'surveying techniques,' skimming the material, as a way to start studying and build a foundation. Number of organizational techniques recommended. For studying, suggests concept mapping and highlighting, creating questions based on the material, imaging and visualization. Regarding group studying: efficient method of reinforcement, a way to take a step further from memorization to conceptual understanding. |
| Anonymous, n.d.  Australia [22] | To provide tips for studying in medical school | Opinion:  Article on general practice student support website | Physicians and general practice residents in Australia contributed, number of contributors unknown | Tips: stay organized, focus on the big picture, know the terminology and nomenclature, compare and contrast topics, study every day, study in groups, ask questions, translate notes you receive into your own version, avoid excessive caffeine, try to enjoy what you are dong |
| Anonymous, 2007  USA & Canada [23] | To discuss learning strategies and what works for students | Opinion:  Online discussion forum | 26 (medical students, resident trainees, practicing physicians and unspecified) | Medical trainees' perspectives on study groups vary significantly. Some find solitary self-study to be the most effective due to efficiency, and feel that group studying can cause additional stress over not being as prepared as his/her peers. Advantages to group study: like-minded individuals work well together, peer motivation, ability to discuss cases, quiz each other, and solidify information, diffusing the amount of work, mutual accountability. Some describe studying in the presence of peers, without actually studying together, is motivating and more effective, and allows them to ask each other questions when they need. |
| Anonymous, n.d.  USA [20] | To share student experiences and ideas regarding medical school | Opinion:  Article on school website | 4 medical students | Expressed value of combining both solitary study and group study. Describe writing notes independently to learn the material for the first time, and then using study groups to review material. One student described that study groups reinforce whether or not she knows the material. Another student describes that study groups are most useful to her a day or two before the exam, and that asking questions back and forth is helpful. The third student doesn't use study groups, but likes to study in the same room as other students. Results include other recommendations regarding keeping up with workload, organizational systems, maintaining balance, getting value out of lectures and textbooks, and other study tips. |
| Boysen *et al.,* 2016  USA [27] | To describe the differences between generations and the impact of this on medical education | Descriptive:  Literature review and commentary | Not applicable – personal commentary | Generation 2020 prefers social learning and recent technology developments enable peer-to-peer learning. Lecture attendance often poor despite high USLME scores, with peer-to-peer learning occurring outside these lectures more appreciated and perceived as effective. |
| Brandt *et al.,* 2011  USA [24] | To provide new medical students with a guide on how to study | Opinion:  Blog article | Not applicable – personal commentary | There are many different strategies and some will work for some and others for others. Techniques for active studying differ from techniques for active reviewing. Group discussion, quizzing and peer tutoring are better designed for review. Students should be open to evolution of study strategies based on what works and doesn’t work. Non-science majors may benefit from linking up with science-major classmates to explain topics to them and help them learn to recognize the high yield material. |
| Burford, 2012  England [45] | To introduce social identity approach from social psychology to medical education research and to illustrate how the approach may afford 'better ways' of thinking about social identity in medical school | Descriptive:  Social identity approach to research described in context of tis relevance to medical education research | Not applicable - personal commentary | Social Identity approach may inform the achievement of professional identify formation in medicine, which involves exploration of 'fit' within medicine as expressed by others. Second area that may be illuminated by social identity theory is inter-professional and multidisciplinary team-working. ion within groups. A role for social identity theory in evaluating communication and assessment validity issues in medicine is described. |
| Chou *et al.,* 2014  USA [32] | To investigate the role of peer groups (both formal and informal) on student learning during clerkship | Survey | Third year medical students halfway through their core clerkship year. 29 students in clerkship models with continuity, and 44 students in traditional block clerkships. | Almost all students described their self-identified peer groups as sources of social and academic support. Students in continuing medical curricula valued regular meetings and identified the value of checking in with their peers to help each other navigate issues in clerkship. Traditional curricula students tended to meet on an ad hoc basis and valued these opportunities. Academic support themes: assistance with study skills, sharing learning materials, transitioning to new clinical experiences. Peer groups in both settings oriented students to the culture of clinical settings/teams and to the expectations. A minority of students identified their peers from the first two years of medical school to be more helpful in understanding their roles/responsibilities in clerkship. |
| DeVoe *et al.,* 2007  USA [34] | To describe the study group program implemented at the University of New Mexico School of Medicine, present the results, and offer recommendations based on the experience | Experiment:  Pilot intervention of study groups led by senior medical students for medical students considered to be ‘at risk’ | 13 first year medical students | No significant differences in scores were seen between matched groups. Points that came up from the questionnaires given to students were as follows: self-selection into study groups was preferred over mandated participation; participants thought the groups helped them adjust to medical school more than academically. Problems raised were that mandatory participation threatened motivation and, as such, giving students the ability to organize and lead these groups on their own may be better. |
| Dick, 2012  UK [28] | To determine the role of medical students' peers in academic support in medical education | Descriptive:  Case study with individual, semi-structured interviews | Nine medical students who failed a minimum of one summative examination, and eight faculty educators involved in provision of student support in medical school | (1) Senior peers provide reassurance and practical advice in preparation for a resit examination; (2) peer support helps individuals to gauge their level of knowledge in relation to the required standard; (3) Same year peers who have failed help to reduce the burden of a resit examination; (4) same year peers who have failed help each other augment learning in preparation for a resit examination; (5) failing an exam reduces the need to impress peers |
| Ding, 2015  USA [39] | To provide opinions from former med-student to current med-student on studying | Opinion:  Blog article with discussion thread | Not applicable for blog (personal commentary), but ten members of discussion thread, some of which self-identify as medical students while others do not make their educational status known. Author of blog is a practicing physician. | Six recommendations were made: (1) skip class to save time; (2) don't use textbooks but, rather, lecture notes; (3) make your own notes; (4) use technology (organization + efficiency); (5) study individually, as group study - though perhaps more comprehensive - is less time efficient; (6) do practice questions to test yourself |
| Hafferty *et al.,* 2013  USA [3] | To better understand the "networked" life of the medical school as a learning environment | Survey | 48 first-year medical students | An increase in network connectivity was seen over time. Some social hubs emerged in regard to out-of-class questions, general curricular issues and professionalism role modelling. Hubs regarding initial group formation and informal group formation centred around students who did not do very well academically but were regarded highly by peers and faculty. With respect to peer assignment of professional role-model status, the central student was both academically strong and well regarded, but was not considered a "go-to" person for peers regarding academic questions. The class became more informally networked over time. In all regards, one's formal group assignment was shown to have an initial impact but one that decreased over time, with informal student network gaining importance. Links were seen based on gender. There did not appear to be ethnic-minority exclusion from groups, or cliquing, but there was an under-representation of these minorities in the largest network component. |
| Hendry *et al.,* 2005  Australia [19] | To measure the nature of student study groups - factors important in their formation, how they function and what students perceive as the advantages and disadvantages of study group membership | Survey | 233 year 2 medical students | Length of time study groups stayed together was positively related to achievement in the written summative assessment examined. No difference in assessment scores between those who were and were not in a study group. Students formed groups mostly because group members were their friends and had similar personalities to their own. Advantages described: motivation, emotional support, clarification of concepts. Disadvantages include extra time required to prepare for meetings, wasting time socializing, and large group size. |
| Kingsbury, 2014  UK [17] | To provide the benefits and drawbacks of informal study groups in medical school | Opinion:  Blog article | Not applicable – personal commentary | Pros: sharing knowledge, keeping learning engaging, socialization for future teamwork in medicine. Cons: potential for socialization to be distracting, unproductive without a plan, can be social discrepancies and forced leadership. |
| Kingsbury, 2014  UK [30] | To provide advice new medical students re how to study in medical school | Opinion:  Blog article | Not applicable – personal commentary | Establish study location and time frame, actively engage with the reading (taking notes/summarizing), study in groups to quiz each other and fill in gaps in knowledge, use healthy and energizing rewards to motivate you to keep studying |
| Kulac *et al.,* 2015  Turkey [42] | To determine the influence of learning styles and gender on success in medical school | Survey | 282 medical students in years 1-3 (preclinical stage) | Competitive and collaborative were the most frequent learning styles demonstrated by the medical students included in the study. Competitive learning was found to be associated with higher mean final exam scores. Female students scored significantly higher than males when competitive learners. Females with collaborative style had significantly higher block exam scores than males with collaborative learning styles. Independent learners represented the smallest portion of the study sample. |
| Lovell, 2015  UK [1] | (1) To determine how medical students perceive the nature and purpose of medical social groups; (2) To determine the factors that influence the creation and maintenance of these communities | Descriptive:  Semi-structured interviews according to constructivist grounded theory approach | 32 final-year medical students from two London medical schools | Four major themes identified: (1) enforced isolation, (2) self-isolation, (3) supportive network, (4) judging self and others. Medical students form communities due to both external factors (geographical location) and individual purpose (similar interests, demands). Medical students place great value on the supportive nature of their community; learning found to take place between students in informal settings in the context of revision sessions, study groups, informal journal clubs and other opportunistic teaching in social settings. Moral obligation described to help peers in academic matters. Peers influence each other's study habits as well; students found to critique their own learning styles and motivation and dedication in comparison to their peers. |
| McNeill *et al.,* 2014  Australia [35] | To determine the relationship between medical student group membership and wellbeing and to determine whether group membership was related to unhealthy behaviour among medical students | Survey | 92 medical students from 3 medical schools | Student group/social identification significantly positively affects student well-being. With regards to unhealthy behaviour, a reluctance to seek help norm within groups was related to decreased wellbeing, though this was not true for a group partying norm. |
| Medoff, n.d.  USA [26] | To provide medical students with strategies to improve their study skills | Opinion:  Article on medical school website | Not applicable – personal commentary | Tips: use practice exams/study aids, read actively, prepare in advance for lectures, focus during lectures, review within a day or two of the lecture, study in an appropriate atmosphere for you. Regarding social studying: works for some and not others, study with people who are different from you and can provide different perspectives, and agree on the goals/norms of the group. Also addresses listening to your body, staying healthy, and understanding your own learning style. |
| Petersen, 2012  USA [16] | To guide medical students regarding the best way to utilize study groups | Opinion:  Blog article | Not applicable – personal commentary | 3-4 students recommended as ideal group size. Setting an agenda, preparing for sessions, and scheduling a specific time period in advance were recommended. Taking turns teaching and learning from each other was also recommended. The three main goals addressed were to (1) emerge from a session with a list of what you don't know yet, (2) determine what you already know well, and (3) personalize and interact with the material. |
| Riddell *et al.,* 2016  UK [41] | To provide an argument supporting near-peer education as an addition to traditional teaching in medicine. | Opinion:  Letters to the editor | Not applicable – personal commentary | Medical students view opportunities to learn from their peers as highly valuable |
| Schauber *et al.,* 2015  Germany [43] | To determine the relations between educational outcomes, emotions, appraisals, and aspects of the environment (social support) in both problem-based-learning (PBL)-focused and traditional medical curriculums (TMCs). | Survey | 1646 medical students | PBL curricula tend to be associated with better student perceptions of the learning environment. Insignificant difference in academic achievement (small advantage to TMC students). Gains in achievement in both curricula are related to self-study effort, including beneficial approaches to learning such as collaborative studying or metacognitive approaches to learning. Engaging in collaborative studying shown to have benefits in terms of learning and perceived social support among students, which is related to higher self-efficacy beliefs and a more positive perception of the learning environment. |
| Scott *et al.,* 2014  UK [37] | To describe the development of peer-mediated learning through student-focused and student-led study groups, termed 'Shadow Modules' | Descriptive:  Collaborative learning program and results of its implementation described | Not applicable – descriptive article on component of a medical school program | Peer-mediated learning and collaborative learning useful in anatomy teaching; fosters practice in learning and promotes deep learning approaches. The student-focused and student-led 'Shadow Modules' described take place parallel to the formal academic teaching to facilitate collaboration between students. Students collaborate towards curating existing online open resources and develop resources of their own to support their study. These tools are shared online between peers. Specifically, the study found that collaborative learning was enhanced and more favourable when lecturers were not present in the meetings. Feedback from students showed that collaboration with peers in the shadow module made studying more time effective. The authors explain that students readily form small communities of practice in learning on their own but these group sessions have the potential to be much further-reaching. |
| Taylor, n.d.  Unspecified region [18] | To outline advantages and disadvantages of study groups | Opinion:  Website article | Not applicable – personal commentary | Studying in groups allows information sharing, efficiency, shared problem solving, and motivation. Author highlights benefits of repetition, and describes particular benefit of study groups in medical school because of the large volume of information to be memorized and, in particular, in anatomy learning. Disadvantages of studying in a group are addressed, including group members not doing their part and group distraction. |
| Vaughan *et al,* 2015  UK [5] | To determine the impact of relationships on medical student achievement by ethnicity | Survey | 158 medical students across 4 hospital placement sites, during their clinical phase | In their self-selected personal academic support (PAS) networks, average of 8 individuals with whom they interacted with in 'activities important to their academic success'. Homophilous by ethnicity, particularly white students. Ethnic homophily was not related to examination achievement. No relationship between size or interconnectedness of students' PAS networks and exam scores. Students with non-White backgrounds more often achieved lower grades. Muslim students had a lower mean rank and were more likely to appear in the lowest academic quartile. Network factors had greater impact on achievement, though, than ethnicity or religion. |
| Woolf *et al.,* 2013  UK [36] | To investigate whether demographic and psychological factors mediate the relationship between ethnicity and final examination scores | Survey | 703 medical students | Univariate ethnic differences were found on age, personality, learning styles, living at home, first language, parental factors, and prior education. Minority ethnic students had lower final exam scores, were more likely to fail, and less likely to achieve a merit or distinction in finals. Multivariate analyses showed ethnicity predicted final exam scores even after taking into account questionnaire factors. |
| Woolf *et al.,* 2012  UK [2] | To measure the formation of medical student social networks and their relationship to grades | Survey | 215 medical students | Students of same sex, same ethnic group and same tutor and small groups (to which randomly assigned) were socially closer. Students' choice of friends had a significant influence on their examination grades, even after taking their previous grades into account. I.e. students doing well were more closely linked socially to other students who were also doing well, and students who were performing poorly were more closely linked socially to other students who were also performing poorly. |
