# Supplementary material for: Social studying and learning among medical students: a scoping review
Source: Perspect Med Educ. 2017 May 17;6(5):311–8. doi: 10.1007/s40037-017-0358-9 (PMC5630528; doi:10.1007/s40037-017-0358-9)
Supplement: Supplementary file 2 — Characteristics of the reviewed sources [file 40037_2017_358_MOESM2_ESM.docx]

|  | Peer-Reviewed Published Literature (N=18) – number (%) | Grey Literature (N=17) – number (%) |
| --- | --- | --- |
| Source Type | | |
| Descriptive | 3 (16.7) | 0 (0.0) |
| Interviews and/or focus groups | 3 (16.7) | 0 (0.0) |
| Survey | 10 (55.6) | 0 (0.0) |
| Experiment | 1 (5.6) | 0 (0.0) |
| Social Media/Online Forum | N/A | 3 (17.5) |
| Opinion | 1 (5.6) | 14 (82.4) |
| Student Voices in Data | | |
| Exclusively | 4 (22.2) | 3 (17.6) |
| Extensively | 6 (33.3) | 3 (17.6) |
| Minimally | 5 (27.8) | 1 (5.9) |
| None | 3 (16.7) | 9 (53.0) |
| Unknown | 0 (0.0) | 1 (5.9) |
| Author Type | | |
| Medical student | 3 (16.7) | 5 (29.4) |
| Practicing physician | 5 (27.8) | 6 (35.3) |
| Researcher | 15 (83.3) | N/A |
| Educator | 1 (5.6) | 3 (17.6) |
| Resident (trainee) | 0 (0.0%) | 4 (23.5) |
| Unknown | N/A | 7 (41.2) |

*Table 2: Characteristics of the reviewed sources*
